# DNA Oxidative Cleavage Induced by the Novel Peptide Derivatives of 3-(quinoxalin-6-yl)alanine in Combination with Cu(II) or Fe(II) Ions

**DOI:** 10.1155/2009/906836

**Published:** 2010-03-08

**Authors:** Wojciech Szczepanik, Marzena Kucharczyk-Klamińska, Piotr Stefanowicz, Anna Staszewska, Zbigniew Szewczuk, Jacek Skała, Andrzej Mysiak, Małgorzata Jeżowska-Bojczuk

**Affiliations:** ^1^Faculty of Chemistry, University of Wrocław, Joliot-Curie 14, 50-383 Wrocław, Poland; ^2^Institute of Biochemistry and Biophysics, Polish Academy of Sciences, Pawińskiego 5a, 02-106 Warsaw, Poland; ^3^Microbiological Institute, University of Wrocław, Przybyszewskiego 63, 51-148 Wrocław, Poland; ^4^Medical University of Wrocław, Pasteura 4, 50-367 Wrocław, Poland

## Abstract

Three model dipeptides containing 3-(2,3-di(pyridin-2-yl)quinoxalin-6-yl)alanine, 3-(dipyrido[3,2-a:2,3-c]phenazin-11-yl)alanine, and 3-(2,3-diphenylquinoxalin-6-yl)alanine were studied with respect to their ability to bind selected transition metal ions, such as Cu(II), Fe(II), Ni(II), Co(II), Mn(II), and Cr(III). It was found that only Cu(II) and Fe(II) ions could form stable complex species with the studied compounds. The ability to form the complexes correlated well with DNA damage experiments. Only the ferrous and cupric complexes are capable of generating both single- and double-strand scissions. However, double-strand breakages appear to be dominating lesions in the presence of hydrogen peroxide, especially for copper(II) containing systems. The quantity of breakage products in the presence of *N*-(3-(dipyrido[3,2-*a*:2,3-*c*]phenazine-11-yl)alanyl)glycine complexes was the highest as compared to the complexes of the remaining compounds. Moreover, this ligand was the only one that cleaved DNA in the absence of either Cu(II) or Fe(II) ions.

## 1. Introduction

Small molecules binding to specific sites along DNA molecule are considered as potential chemotherapeutic agents. Numerous examples of such systems include Cu(II) complexes containing heterocyclic bases. Sigman et al. [1–4] have discovered that in the presence of H_2_O_2_ and a reducing agent the (Phen)_2_Cu(II) complex exhibits an efficient DNA cleavage activity. In order to increase the selectivity towards the defined sequences of nucleic acids the active complex may be conjugated with a complementary RNA sequence [5–7], PNA [8], or polypeptide [9] showing affinity to a defined sequence of nucleic acids. This approach may be applied for designing antiviral drugs [10]. 

 Many DNA interactive heterocyclic compounds, including some quinoxaline derivatives, have been already evaluated. Among these, 2,3-di(pyridin-2-yl)quinoxaline (DPQ) and dipyrido[3,2-a:2,3-c]phenazine (DPPZ), complexed with transition metals, are of particular interest in view of their binding to DNA. They are known as “chemical nucleases” that efficiently nick DNA causing oxidative cleavage [11]. It has also been found that the copper(II) complex of DPPZ binds to the DNA minor groove causing hydrolytic cleavage of supercoiled DNA in the dark and in the absence of any external reagents [12]. 

 A series of ruthenium and platinum complexes has been studied in respect of DNA binding [13, 14]. The Pt(II) coordination species have proven their potential therapeutic usefulness by being claimed as ambivalent intercalators of DNA [14]. Other studies on complexes of pyridyl derivatives of quinoxaline with palladium [15], manganese [16], copper [17], or platinum [18] have presented these compounds as highly effective metal chelators that may show significant chemotherapeutic activity. Recently, DPPZ alone has been tested against cancer cell lines. The cytotoxic activity of this compound is generally comparable to that of cisplatin, a widely used anticancer drug, however in some cell lines (e.g., A2780R) the activity of DPPZ is significantly higher [19]. 

 It may be expected that peptides conjugated with quinoxaline analogs will help design new DNA targeting agents. Attachment of the peptidic chain to a potentially biologically active substance may enable its cellular recognition and subsequent transport. The literature provides numerous examples of targeting achieved by adding ligand moiety to a biologically active molecule (e.g., adhesive RGD sequence [20], LHR peptide [21], or PEGA peptides [22]) directed to certain types of binding sites. This approach is often used in cancer therapy, as it significantly reduces the cytotoxic effect on healthy organs. 

**Figure 1:**
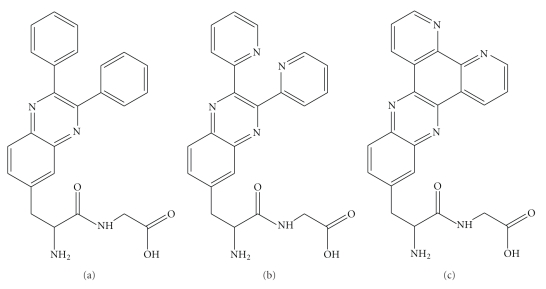
Structures of the studied compounds: *N*-(3-(2,3-diphenylquinoxalin-6-yl)alanyl)glycine (DFQa-Gly, (a)), *N*-(3-(2,3-di(pyridin-2-yl)quinoxalin-6-yl)alanyl)glycine (DPQa-Gly, (b)) and *N*-(3-(dipyrido[3,2-*a*:2,3-*c*]phenazine-11-yl)alanyl)glycine (DPPZa-Gly, (c)).

 Recently, we have developed an efficient method of solid-phase synthesis of peptides containing quinoxaline-derived amino acid side chains [23]. The synthesized dipeptides contain various analogues of 3-(quinoxalin-6-yl)alanine, including quinoxaline derivative of 2,3-bis(2-pyridyl) quinoxaline (DPQ), dipyrido[3,2-a:2,3-c]phenazine (DPPZ), and 2,3-bis(2-phenyl)quinoxaline (DFQ) (Figure 1). 

 In that paper we have determined whether quinoxaline analogues directly conjugated to the side chain of a model peptide cleave DNA in a similar manner as the corresponding quinoxaline analogues alone [11, 12]. The possibility to preserve the properties of chemical nuclease following conjugation to the peptide molecule by the studied heterocyclic compounds opens new perspectives for designing molecules targeting defined tissues.

## 2. Experimental Section

### 2.1. Materials

CuCl_2_, FeCl_2_× 4H_2_O, KH_2_PO_4_, K_2_HPO_4_, acetonitrile, ammonium acetate, H_2_O_2_, ascorbic acid, bromophenol blue, glycerin, agarose, and all other simple chemicals were purchased from Sigma-Aldrich Chemie. All peptide derivatives solutions were purified from the trace amounts of redox-active metal ions by adding Chelex-100 resin.

### 2.2. Synthesis of Peptide Derivatives

Peptides were synthesized on the solid support according to the method described recently [23].

### 2.3. Preparation of DNA

pBluescriptSK+ plasmid DNA was isolated from bacterial cells using Nucleobond AX100 cartridges and buffers (Macherey-Nagel, Duren, Germany, cat. no. 740 573). Bacterial strain was cultivated with vigorous shaking (197 rpm) at 37°C in 100 mL of LB medium (1% yeast extract), 1% bactotryptone, 0.5% sodium chloride, and 100 *μ*g/mL ampicillin until the mid-logarithmic growth phase. Cells were harvested by centrifugation (5000 × g, 10 minutes, 4°C) and resuspended in 4 mL of S1 buffer (50 mM Tris–HCl, pH 8.0; 10 mM EDTA; 100 mg/mL RNase A). Then 4 mL of S2 buffer (200 mM NaOH; 1% SDS) were added, and cell suspension was gently mixed and incubated for 5 minutes at room temperature. Cell lysate was then mixed with 4 mL of S3 buffer (2.8 M sodium acetate, pH 5.1), incubated for 5 minutes on ice and clarified by filtration. Finally, the cell lysate was loaded on a Nucleobond AX100 column equilibrated with N2 buffer (100 mM Tris–H_3_PO_4_, pH 6.3; 15% ethanol, 900 mM KCl). After washing with 8 mL of N3 buffer (100 mM Tris–H_3_PO_4_, pH 6.3; 15% ethanol, 1150 mM KCl) plasmid DNA was eluted from the column using 4 mL of N5 buffer (100 mM Tris–H_3_PO_4_, pH 8.5; 15% ethanol, 1 M KCl). Finally, DNA was recovered from buffer N5 by isopropanol precipitation and redissolved in 100 *μ*L of 10 mM Tris–HCl, pH 8.0.

### 2.4. DNA-Strand Break Analysis

The ability to induce strand breaks by the studied ligands and their complexes with metal ions in the presence and absence of either H_2_O_2_ or ascorbate was tested with the pBluescriptSK+ plasmid. Similar method has been previously applied to the investigations of DNA cleavage by the range of copper(II) and nickel(II) complexes [24–26]. The buffered samples (phosphate buffer, pH 7.4) contained combinations of DNA (25 *μ*g/mL), and the components of the investigated systems (metal ion, ligand, and H_2_O_2_ or ascorbate) at the concentration of 50 *μ*M. In the preliminary experiments MnCl_2_, Co(NO_3_)_2_, CrCl_3_, and NiCl_2_ salts were additionally applied also at the concentration of 50 *μ*M. 

 After 1 h of incubation at 37°C (except the kinetic measurements), the reaction mixtures (20 *μ*L) were mixed with 4 *μ*L of loading buffer (bromophenol blue in 30% glycerol) and loaded on 1% agarose gels, containing ethidium bromide, in TBE buffer (90 mM Tris-borate, pH 8.0; 20 mM EDTA). Only in the experiment aimed to compare the DNA retarding abilities of the studied substances the gel was stained with ethidium bromide after the separation. Gel electrophoresis was done at a constant voltage of 4 V/cm for 60 minutes. As a control for double-strand breaks, reference plasmid samples were linearized with *Eco*RI endonuclease. The gels were photographed and processed with a Digital Imaging System (Syngen Biotech, Wrocław, Poland).

### 2.5. ESI-MS Analysis of Complexes

High-resolution mass spectra were obtained on a Bruker MicrOTOF-Q spectrometer (Bruker Daltonik, Bremen, Germany), equipped with an Apollo II electrospray ionisation source with an ion tunnel. The mass spectrometer was operated in the positive ion mode. The instrumental parameters were as follows: scan range m/z 400–1600, dry gas-nitrogen, temperature 200°C, ion source energy 5 eV, collision energy 10 eV. The dipeptides reconstituted with 0.5 or 1 equivalent of Cu(II) or Fe(II) ions, respectively, were dissolved in 10 mM ammonium acetate in water/acetonitrile (1 : 1). Although similar MS spectra were obtained both in water and in water/acetonitrile solution, the peak intensities were significantly lower in water. This did not allow to obtain spectra with a sufficient signal-to-noise ratio. Therefore, we decided to present spectra in water/acetonitrile solution (1 : 1) only. The solutions (0.1 mM in respect to peptide) were infused at a flow rate of 3 mL/minutes at room temperature. Before each run the instrument was calibrated externally with the Tunemix mixture (Bruker Daltonik, Germany) in the quadratic regression mode. The mass accuracy of the calibration was 5 ppm or better, which together with the true isotopic pattern (using SigmaFit) enabled an unambiguous confirmation of the elemental composition of the obtained complexes.

### 2.6. Electronic Absorption Spectroscopy

The electronic absorption (UV-Vis) spectra were recorded on a Varian Cary 50 Bio (Varian, Palo Alto, CA) spectrophotometer over the spectral range 300–800 nm, in a 1 cm cell at 25°C. For complexation experiments, aqueous samples with the 1 : 1 and 1 : 2 metal-to-ligand molar ratios were used, with the Cu(II) concentration of 1 mM. Due to the presence of vestigial amounts of copper(II) hydroxide in the equimolar system, only the spectra for the systems, where M/L = 1 : 2 were analysed. Fe(II) complexes of the studied compounds were investigated at the Fe(II) concentration of 1 mM and the M/L ratio 1 : 3 but no measurable transitions were detected. The pH of the samples was adjusted to 7.4 with small amounts of concentrated NaOH.

### 2.7. Circular and Magnetic Circular Dichroism Spectroscopy

CD and MCD spectra were recorded at 25°C on a Jasco J-715 spectropolarimeter (JASCO, Japan Spectroscopic Co., Hiroshima, Japan), over the spectral range of 200–800 nm, using a 1.0 and 0.1 cm cell. Samples were prepared using distilled and demineralised water at the 1 : 2 metal-to-ligand molar ratio for the Cu(II) complexes and 1 : 3 for Fe(II) species, with the metal concentration of 1 mM. The solutions of FeCl_2_ were protected from air and the preparation of the iron complexes was carried in the argon atmosphere in the glovebox. The pH of the sample was adjusted to 7.4 with small amounts of concentrated NaOH. The magnetic field used in the MCD measurement was 1.47 T.

## 3. Results and Discussion

### 3.1. Complex Formation

The following combinations of peptides: DPQa-Gly, DFQa-Gly, DPPZa-Gly, and metal ions: Cu(II) or Fe(II), were studied by ESI-MS, which is known as a rewarding method to investigate noncovalent interactions [27]. Many recent applications of this soft ionisation technique suggest that gas phase data reflect, to some extent, the interactions occurring in solution [28]. This method is also established in the studies of metal-protein and metal-peptide interactions, providing information on the stoichiometry of complexes [29], and to a certain degree, the localization of binding sites [30, 31]. 

**Figure 2:**
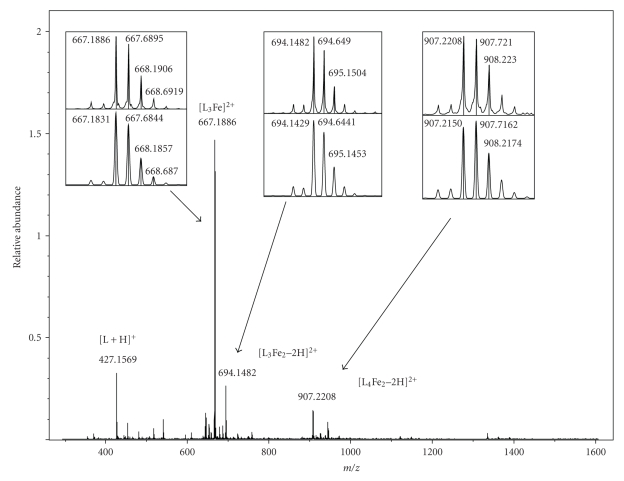
ESI-MS spectrum of the complexes formed by DPPZa-Gly and Fe(II). Concentrations of ligand and Fe(II) ions were 0.1 mM in the water/acetonitrile mixture (1 : 1) containing 10 mM of ammonium acetate.

 In the ESI-MS experiment (Table 1), the stoichiometry of the analysed complexes does not significantly depend on the ligand-to-metal molar ratio in solution (in the range 1 : 1 to 1 : 2). However, at the 1 : 1 ratio, we obtained slightly larger signals from the complexes of DPPZa-Gly and DPQa-Gly with Fe(II) and/or Cu(II) than at the 1 : 2 ratio. The experimental isotopic patterns of the positively charged complexes were in perfect agreement with the simulated isotopic pattern distributions (Figure 2). The highest abundance of complex ions was detected for the DPPZa-Gly peptide. The predominant type of complexes with Cu(II) was [L_2_Cu_2_–2H]^2+^, while the major form with Fe(II) ions was [L_3_Fe]^2+^. However, a careful analysis of the isotopic patterns of side peaks revealed the presence of some amounts of [L_3_Fe_2_–2H]^2+^ and [L_4_Fe_2_–2H]^2+^complexes (Figure 2). The predominant type of complex of DPQa-Gly with a Cu(II) ion was [LCu–H]^+^; whereas [L_2_Cu_2_–2H]^2+^ was observed as a minor species (data not shown). In the same conditions, no signals corresponding to DFQa-Gly complexes were observed, which might indicate that either peptide DFQa-Gly did not form complexes with Cu(II) or with Fe(II) ions, or that the complexes formed were not sufficiently stable under ionisation conditions. These results may suggest that the structure of the heterocyclic part of the molecule is crucial for the stability of the formed complexes. Only these ligands which contain two adjacent nitrogen atoms in positions suitable for chelation of the metal ion show a strong affinity for Cu(II) and Fe(II) ions. The highest affinity was observed for DPPZa-Gly, a compound containing the substructure of 1,10-phenanthroline. The orientation of heterocyclic nitrogen atoms in this rigid ligand is preferable for complexation of metal ions. 1,10-phenanthroline and related compounds are well-known chelating agents for metal ions, including Cu(II) and Fe(II) [32]. The increased affinity of DPQa-Gly and DPPZa-Gly as compared with DFQa-Gly for Cu(II) and Fe(II) ions suggests that the two heterocyclic nitrogen atoms are responsible for the complexing. The composition and structure of the [L_3_Fe]^2+^ complex are the same as in the case of the Fe(II)-phenanthroline complex with six nitrogen atoms distributed in the octahedral way. 

 The three studied ligands accompanied by Cu(II) ions give absorption spectra with transitions at around 620 nm (Table 2). Since the maximum of the Cu(II) aqua complex absorption is located at *ca*. 800 nm, such a distinct shift of the band maximum and an increase of its intensity prove that the coordination between Cu(II) ions and the peptides is favoured. The Cu(II) complex with DFQa-Gly was not observed in the spectra obtained by ESI-MS. However, its spectra were recorded in solution using UV-Vis spectroscopy. A lower value of the molar coefficient in the case of Cu(II)–DFQa-Gly complex (48 M^−1^ cm^−1^) in comparison with the remaining two systems may indicate its lower stability. The CD spectroscopy was also applied to verify the interaction between Cu(II) ion and the studied compounds. Despite the relative similarities in the UV region, there is a visible range of a different course of d-d transition bands, which suggests dissimilar coordination surroundings in all three cases; see Figure 1 in Supplementary Material available online at doi:10.1155/2009/906836.

 The UV-Vis spectroscopy performed for the systems with Fe(II) and the studied peptides showed a very weak band between 930 and 960 nm. This transition is typical of the high spin octahedral Fe(II) species; however its quantitative analysis was not possible due to very low absorption. To confirm the complex formation in those cases, MCD spectroscopy was applied. Only Fe(II)–DPPZa-Gly system gave a legible spectrum with transitions at 324, 380, 425, 510, and 530 nm (see Figure 2 in Supplementary Material). Unlike the complex, the uncomplexed Fe(II) ions and the free ligand gave very poor MCD spectra. The bands at 324 and 380 nm originate from the intraligand transitions, the one at 425 nm is most probably the charge transfer transition, while both 510 and 530 nm bands are typical of iron(II) complexes [33]. The spectrum obtained for the Fe(II)–DPQa-Gly system is less informative, while the Fe(II)–DFQa-Gly complex gave no MCD spectra at all, potentially due to its low stability.

### 3.2. DNA Cleavage

DNA breakage studies constitute a useful tool to estimate potential procarcinogenic or promutagenic properties of molecules, especially those that are suspected to reveal therapeutic activity. The quinoxaline derivatives, DPQa-Gly, DPPZa-Gly, and DFQa-Gly, as well as their Cu(II) and Fe(II) complexes were screened for the DNA cleaving properties with the use of gel electrophoresis. Two types of DNA scission could be observed in these systems provided that plasmid was used as a reference DNA molecule. It allows for easy recognition of both single- and double-strand damage that leads to formation of the nicked/relaxed plasmid (form II) and the linear DNA (form III). These two products may also be effectively separated from the substrate, that is, the superhelical plasmid (form I). 

**Figure 3:**
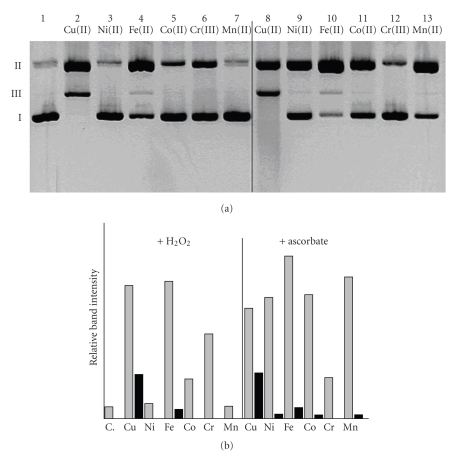
(a) Pattern of the plasmid DNA damage by DPQa-Gly complexes with selected transition metal ions in the presence of H_2_O_2_ (lanes 2–7) and ascorbate (lanes 8–13). Untreated DNA, lane 1; (b) Densitogram of the gel presented in Figure 3(a); grey bars represent relative amounts of the nicked plasmid (form II), black bars represent relative amounts of linear plasmid (form III).

 The target of the preliminary experiment was to select such metal ions that may affect the reactivity of the studied molecules. Six ions were chosen taking into account their ability to form stable complexes with ligands rich in nitrogen donors, as well as the ability to undergo oxidative reactions. Figure 3(a) presents the results of the study, where Cu(II), Ni(II), Fe(II), Co(II), Cr(III), and Mn(II) ions were used in the presence of hydrogen peroxide, which is an endogenous substance. It may be clearly seen that only Cu(II) and Fe(II) may significantly trigger off the DNA damage in the presence of DPQa-Gly. This is supported by the relatively high amounts of plasmid form II, which results from the single strand damage (Figure 3(b)). Additionally, in both cases plasmid form III is present. This was observed in the presence of either hydrogen peroxide or ascorbic acid. In the absence of any of the red-ox agents scarcely any DNA degradation was observed (data not shown). This suggests that the studied reactions are accompanied by reactive oxygen species. The most probable active substrate might be the hydroxyl radical that is generated in Fenton-like processes and whose presence has been previously confirmed by us in similar systems [24, 25, 34]. An analogous suggestion has been put forward by Thomas et al., who has confirmed the presence of hydroxyl radicals with the use of DMSO in Cu(II)–DPQ system [35, 36]. 

 In the case of Cu(II), the complex cleavage yield seems to be equal regardless of the presence of H_2_O_2_ or ascorbate (Figure 3(b)). This is also observed in the systems with the Fe(II) complex (lanes 4 and 10, Figure 3(a)). However, the samples which contained Ni(II), Co(II), and Mn(II) complexes accompanied by ascorbate showed a slightly different DNA damage pattern (lanes 9, 11, and 13, Figure 3(a)), when compared to the analogous system with H_2_O_2_ (lanes 3, 5, 7, Figure 3(a)). Ascorbate enhances the yield of cleavage in these systems to a larger extent than hydrogen peroxide, which is confirmed by the increase of form II concentrations in all those cases (Figure 3(b)). Only the samples with Cr(III) do not contain any significant amounts of DNA damage products. This is a probable result of the Cr(III) oxidation to the Cr(VI) species, confirmed by the change of sample colour from light blue to light yellow. 

**Figure 4:**
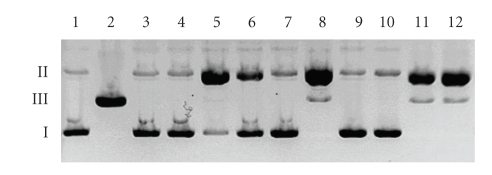
Comparison of the DNA cleavage extent in the presence of Cu(II) complexes of the studied compounds and H_2_O_2_ at the concentration of 50 *μ*M. Lane 1, untreated DNA; lane 2, DNA treated with *Eco*RI endonuclease; lane 3, DNA + DFQa-Gly + H_2_O_2_; lane 4, DNA + Cu–DFQa-Gly; lane 5, DNA + Cu–DFQa-Gly + H_2_O_2_; lane 6, DNA + DPPZa-Gly + H_2_O_2_; lane 7, DNA + Cu–DPPZa-Gly; lane 8, DNA + Cu–DPPZa-Gly + H_2_O_2_; lane 9, DNA + DPQa-Gly + H_2_O_2_; lane 10, DNA + Cu–DPQa-Gly; lane 11, DNA + Cu–DPQa-Gly + H_2_O_2_; lane 12, DNA + Cu + H_2_O_2_.

Figure 4 presents a detailed analysis of plasmid damage by the studied molecules, as well as their Cu(II) complexes accompanied by H_2_O_2_. Lanes 5, 8, and 11 represent the samples containing the complexes and it may clearly be seen that DNA degradation is the highest in those particular cases. This is confirmed by the high concentration of the plasmid form II and the lack of the substrate form I. It is worthy of note that the complexes of DPPZa-Gly and DPQa-Gly (lanes 8 and 11) produce higher amounts of scissions than the complex of DFQa-Gly (lane 5). Which is also interesting, the concentration of cleavage products in the case of Cu(II)–DFQa-Gly system is even lower than in the case of uncomplexed Cu(II) ion (lane 12). The oxidative activity of the ligands in the presence of H_2_O_2_ also reveals certain dissimilarity. Neither DFQa-Gly nor DPQa-Gly generate measurable cleavage product amounts (lanes 3 and 9, Figure 4), however, the presence of DPPZa-Gly leads to 50% conversion of the supercoiled plasmid (form I) to its nicked derivative (form II, lane 6). This behaviour seems to be unique, when compared to the other ligands, what may be a consequence of its structure. The planar and rigid moiety in the side chain of the DPPZa-Gly is similar to the 1,10-phenantroline, which is well known for its DNA binding affinity. 

 Interestingly, the plasmid cleavage yield in the case of the Cu(II)–DPQa-Gly complex is identical as in the case of uncomplexed Cu(II) ions (lanes 11 and 12). However, in the cases of the remaining complexes the yields slightly differ. The DFQa-Gly apparently reduces the activity of the Cu(II)–H_2_O_2_ system (lane 5), while the DPPZa-Gly enhances this process (lane 8). This observation allows us to arrange the studied complexes against free copper(II) in the following order of activity: Cu(II)–DPPZa-Gly > Cu(II)–DPQa-Gly = Cu(II) > Cu(II)–DFQa-Gly. 

**Figure 5:**
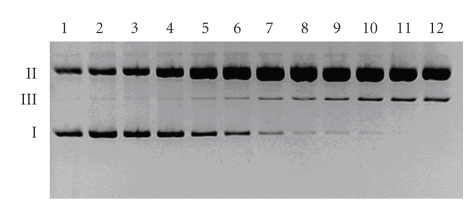
Kinetics of the DNA cleavage in the presence of the Cu(II)–DPPZa-Gly complex accompanied by H_2_O_2_. Incubation periods: 0, 3, 5, 10, 20, 30, 45, 60, 75, 105, 135, and 180 minutes (lanes 1–12, resp.).

**Figure 6:**
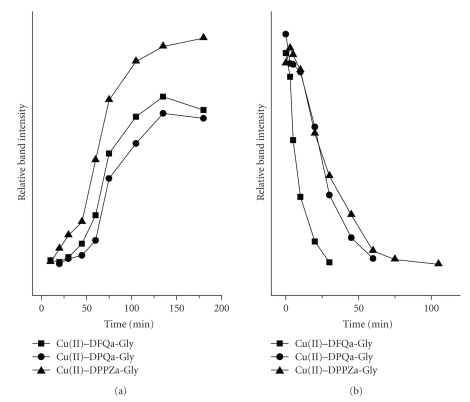
Dependence of the concentration of linearized plasmid (form III, (a)) and superhelical one (form I,(b)) on the progress of reaction presented in Figure 5.

**Figure 7:**
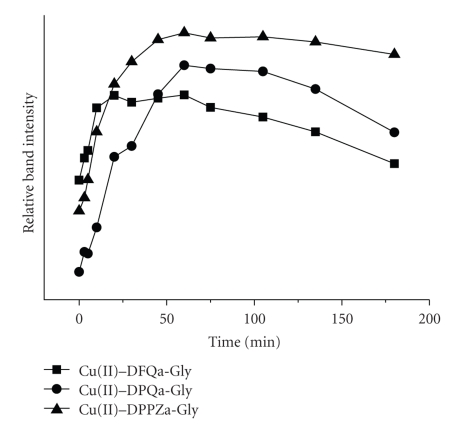
Dependence of the nicked/open plasmid (form II) concentration on the progress of reaction presented in Figure 5.

 In order to investigate whether the double-strand scissions, observed in the presence of the complexes, occur simultaneously at both DNA strands, kinetic experiments were performed. Figure 5 presents the time-dependent mode of the cleavage in the presence of the Cu(II)–DPPZa-Gly complex (for the complexes with DPQa-Gly and DFQa-Gly see Figures 3 and 4 in the Supplementary Material), while Figures 6 and 7 show the quantitative description of the discussed process. It is clear that the kinetic course for each system is similar. In each experiment, measurable amounts of form II are present at the initial step of the reaction. An increase of the form III concentration occurs in a stepwise manner and the highest amounts of form III can be detected in the sample that contains Cu(II)–DPPZa-Gly (Figure 6(a)). The same behaviour was observed in the case of form II. (Figure 7). The pace of its increase in the system containing the Cu(II)–DFQa-Gly complex is slightly higher. However, after approximately 20 minutes. of the experiment, the concentration of form II reaches a *plateau* and after 60 minutes. it drops to the advantage of form III concentration. The yield of the double strand damage in this case is identical as for the Cu(II)–DPQa-Gly system (Figure 6(a)). A decrease of the form I amount in all the studied systems is similar (Figure 6(b)). 

**Figure 8:**
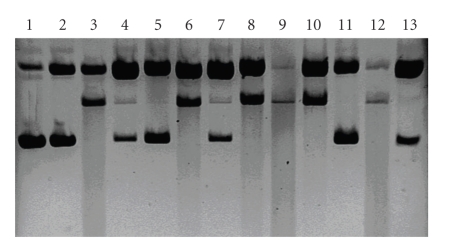
Comparison of the DNA cleavage intensity in the case of both Cu(II) and Fe(II) complexes with the studied compounds in the presence of ascorbate (ASC). Lane 1, untreated DNA; lane 2, DNA + ASC; lane 3, DNA + Cu(II) + ASC; lane 4, DNA + Fe(II) + ASC; lane 5, DNA + DFQa-Gly + ASC; lane 6, DNA + Cu–DFQa-Gly + ASC; lane 7, DNA + Fe–DFQa-Gly + ASC; lane 8, DNA + DPPZa-Gly + ASC; lane 9, DNA + Cu–DPPZa-Gly + ASC; lane 10, DNA + Fe–DPPZa-Gly + ASC; lane 11, DNA + DPQa-Gly + ASC; lane 12, DNA + Cu–DPQa-Gly + ASC; lane 13, DNA + Fe–DPQa-Gly + ASC.

 Following the results obtained from the experiment presented in Figure 3, we also performed a comparative assay for Cu(II) and Fe(II) complexes in the presence of ascorbate. The copper(II) complex with dipyridoquinoxaline has already been proved to show nuclease activity in the presence of ascorbate [11]. This is confirmed by the results from the experiment presented in Figure 8. It is apparent that cupric complexes are more active in the generation of DNA damage than their ferrous counterparts. Smears on the gel that accompany samples containing Cu(II) complexes can be ascribed to the lack of selectivity of the studied systems (lanes 6, 9, and 12, Figure 8). It results in random DNA scission into fragments of various lengths. A similar effect is also observed for Fe(II)–DPPZa-Gly (lane 10, Figure 8), contrary to Fe(II)–DFQa-Gly and Fe(II)–DPQa-Gly treated systems (lanes 7 and 13, Figure 8). This is another evidence for a higher DNA damage by systems containing DPPZa-Gly. 

 There is a more prominent difference in the activity of the Cu(II) aqua complex (lane 3, Figure 8) and Cu(II) complexes with DPPZa-Gly or DPQa-Gly (lanes 9 and 12, resp.) accompanied by ascorbate than it was observed in the experiment with H_2_O_2_. Only Cu(II)–DFQa-Gly cleaves the plasmid in a similar mode and with a similar yield as the Cu(II) aqua complex (lanes 3 and 6, Figure 8). 

 Analysis of the gel presented on Figure 8 shows that ascorbate is more effective agent in promoting DNA damage than H_2_O_2_ possibly because hydrogen peroxide can be produced from ascorbate and Cu(II) in the presence of oxygen [37]. This cooperative process might be an additional source of reactive oxygen species. Similar effects were also observed in the studies on DNA cleavage by the Cu(II) complexes of sinefungin [34] and apramycin [38]. 

**Figure 9:**
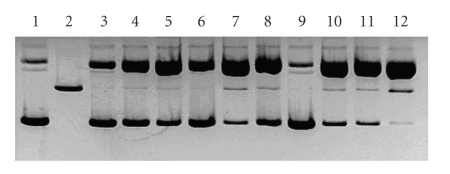
Result of the DNA treatment with the Fe(II) complexes of the studied compounds in the presence and absence of hydrogen peroxide. Lane 1, untreated DNA; lane 2, DNA treated with *Eco*RI endonuclease; lane 3, DNA + DFQa-Gly + H_2_O_2_; lane 4, DNA + Fe–DFQa-Gly; lane 5, DNA + Fe–DFQa-Gly + H_2_O_2_; lane 6, DNA + DPPZa-Gly + H_2_O_2_; lane 7, DNA + Fe–DPPZa-Gly; lane 8, DNA + Fe–DPPZa-Gly + H_2_O_2_; lane 9, DNA + DPQa-Gly + H_2_O_2_; lane 10, DNA + Fe–DPQa-Gly; lane 11, DNA + Fe–DPQa-Gly + H_2_O_2_; lane 12, DNA + Fe(II) + H_2_O_2_.

 Despite the fact that Fe(II) complexes reveal a lower tendency towards DNA damage than the cupric ones, more detailed analysis of their effects is justified by a higher in vivo occurrence of unbound Fe(II) than Cu(II) ions [39]. Figure 9 presents the results of the DNA cleavage experiments for systems containing all the three ligands and H_2_O_2_ in the presence or absence of Fe(II) ions. A comparison of the obtained effects with those for Cu(II) ions in an analogous experiment (Figure 4) shows that Fe(II)–DFQa-Gly is more active in generating scissions than its cupric equivalent (lane 4, Figures 4 and 9). These changes become less apparent following the addition of H_2_O_2_ to the samples. Even more noticeable differences are observed in the case of the Fe(II)–DPPZa-Gly system in the absence of H_2_O_2_ (lane 7, Figures 4 and 9). The Cu(II)–DPPZa-Gly complex does not cleave the plasmid; however, the ferrous analogue is efficient in the generation of single nicks as well as measurable amounts of the double strand scissions (lane 7, Figure 9). The addition of H_2_O_2_ to this system does not influence the yield of the process (lane 8, Figure 9), contrary to the Cu(II)–DPPZa-Gly–H_2_O_2_ that was proved to be the most powerful DNA cleaving agent of all the systems tested (lane 8, Figure 4). Similar results were obtained for the DPQa-Gly complexes with both metal ions. The ferrous species cleaves DNA regardless of the presence of H_2_O_2_ (lanes 10 and 11, Figure 9), while in the case of the Cu(II) complex, H_2_O_2_ determines its activity. 

 Considering the impact of Fe(II) and H_2_O_2_ on DNA in the presence or absence of the ligands, it should be stressed that all three dipeptides reduce the cleavage induced by the Fe(II)–H_2_O_2_ system. The plasmid forms present on the lanes 5 and 8 (Figure 9) clearly show that both DFQa-Gly and DPPZa-Gly restrain the generation of radicals by the metal in similar rate. However, the decrease of plasmid damage in the presence of DPQa-Gly is less apparent than in the former cases, what is supported by the lower concentration of the form I on the lane 11 contrary to the lanes 5 and 8. 

 Summing up the above results, it seems to be of significance that in the presence of either metal ions, or H_2_O_2_, or ascorbate, DPPZa-Gly induces DNA cleavage in the most efficient way among all the studied ligands. Mechanistic investigations using distamycin revealed a minor and a major groove binding for the Cu(II)–DPQ and Cu(II)–DPPZ complexes, respectively, thus suggesting different effects exerted by both quinoxaline derivatives on DNA [29]. On this basis we have compared the influence of DPPZa-Gly (lanes 1–6, see Figure 5 in the Supplementary Material) and DPQa-Gly (lanes 7–12) on the electrophoretic mobility of the plasmid. Various concentrations of both individuals were added to DNA samples. The delay in DNA migration through the gel was observed only in lanes 1–6. This suggests much stronger interaction of DPPZa-Gly with DNA in comparison with DPQa-Gly. Taking the structures of both ligands into consideration, it seems that intercalation is the most likely interaction with DNA. The planar, heterocyclic residue in DPPZa-Gly, similar to 1,10-phenantroline, allows the molecule to intercalate into DNA and thus bind tightly to the nucleic acid. The rotating rings in either DPQa-Gly or DFQa-Gly decrease their affinity to intercalation.

## 4. Conclusions

The results presented above show that DPPZa-Gly, the compound which exhibited the highest activity in DNA cleavage assays, is also a good ligand for both Cu(II) and Fe(II) ions according to the ESI-MS and spectroscopic data. It indicates the importance of the studied peptide modification for the activity of the applied metal ions towards induction of the double-strand scission. A planar quinoxaline residue as well as a peptide moiety responsible for cellular recognition and subsequent transport makes the studied quinoxaline derivatives potent DNA interacting agents. The results indicate the necessity of further exploration of other quinoxaline-containing peptides in terms of their stability, biological transport, and potential intercalating properties.

## Supplementary Material

Figure 1: The CD spectra of the Cu(II) complexed by the studied ligands showing different electronic transitions in each system.
Figure 2: The MCD spectra of the Fe(II) complexes with DPPZa‐Gly and DPQa‐Gly. The DFQa‐Gly ligand does not yield any spectrum with Fe(II) in either CD or MCD spectroscopy.
Figures 3 & 4: The electrophoretic assay analogous to that in Fig. 5 in the manuscript, however with the two remaining ligands used in the reaction mixture.
Figure 5: The assay showing the retarded mobility of the DNA in the samples containing DPPZa-Gly. The possible explanation of this fact might be the intercalation but there is not enough proof.

## Figures and Tables

**Table 1:** ESI-MS characteristics of the complexes of peptides DPQa-Gly, DFQa-Gly, and DPPZa-Gly with Cu(II) and Fe(II) (see Figure 2 for the signals plot).

Peptide	Cu(II)			Fe(II)		
	Relative intensity	*m/z*	Stoichiometry of main complex	Relative intensity	*m/z*	Stoichiometry of main complex
DFQa-Gly	Not detected	—	—	Not detected	—	—
DPQa-Gly	20%	490.0838	[LCu–H] ^+^	Not detected	—	—
DPPZa-Gly	100%	488.0747	[L_2_Cu_2_–2H]^2+^	100%	667.1888	[L_3_Fe]^2+^

**Table 2:** UV-Vis spectroscopic parameters of Cu(II) complexes with DPQa-Gly, DFQa-Gly, and DPPZa-Gly at pH 7.4; ligand-to-metal molar ratio 2 : 1.

Cu(II)–DPQa-Gly		Cu(II)–DFQa-Gly		Cu(II)–DPPZa-Gly	
*λ* [nm]	*ε* [M^−1^cm^−1^]	*λ* [nm]	*ε* [M^−1^cm^−1^]	*λ* [nm]	*ε* [M^−1^cm^−1^]
620	74	620	48	610	90

## Abbreviations

DFQa-Gly: *N*-(3-(2,3-diphenylquinoxalin-6-yl)alanyl) glycine
DPPZa-Gly: *N*-(3-(dipyrido[3,2-*a*:2,3-*c*]phenazine-11-yl)alanyl)glycine
DPQa-Gly: *N*-(3-(2,3-di(pyridin-2-yl)quinoxalin-6-yl) alanyl)glycine.
