# Supplementary material for: DNA Oxidative Cleavage Induced by the Novel Peptide Derivatives of 3-(quinoxalin-6-yl)alanine in Combination with Cu(II) or Fe(II) Ions
Source: Bioinorg Chem Appl. 2010 Mar 8;2009:906836. doi: 10.1155/2009/906836 (PMC2834960; doi:10.1155/2009/906836)
Supplement: Supplementary file 1 — Figure 1: The CD spectra of the Cu(II) complexed by the studied ligands showing different electronic transitions in each system. Figure 2: The MCD spectra of the Fe(II) complexes with DPPZa‐Gly and DPQa‐Gly. The DFQa‐Gly ligand does not yield any spectrum with Fe(II) in either CD or MCD spectroscopy. Figures 3 & 4: The electrophoretic assay analogous to that in Fig. 5 in the manuscript, however with the two remaining ligands used in the reaction mixture. Figure 5: The assay showing the retarded mobility of the DNA in the samples containing DPPZa-Gly. The possible explanation of this fact might be the intercalation but there is not enough proof. [file 906836.f1.pdf]

Supplementary material

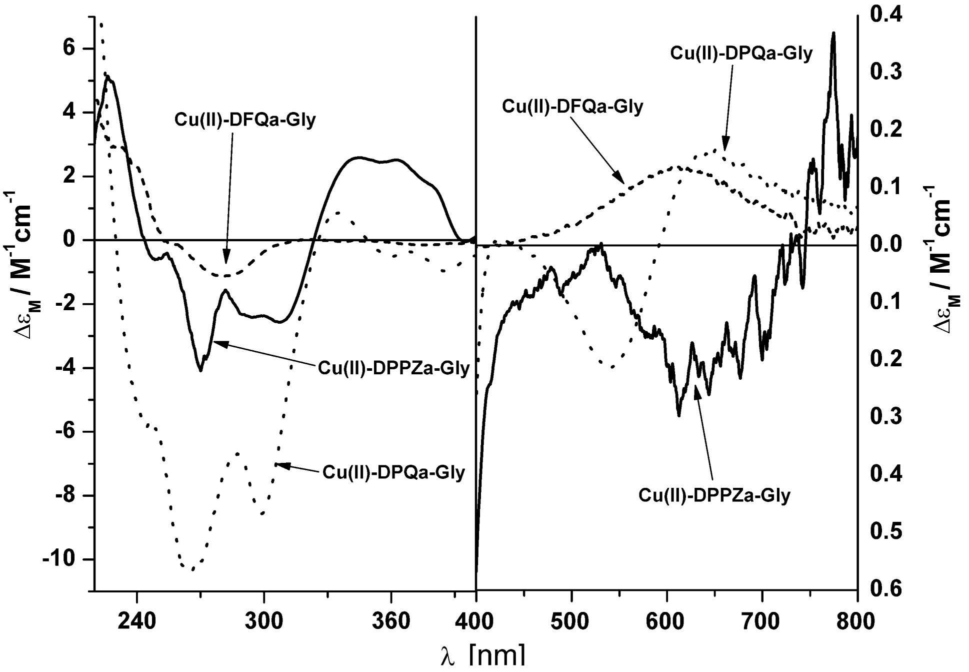

Supplementary Figure 1. Circular dichroism spectra of the Cu(II) complexes with DPPZa-Gly, DPQa-Gly and DFQa-Gly, concentration of metal 1 mM, metal-to-ligand molar ratio 1:2.

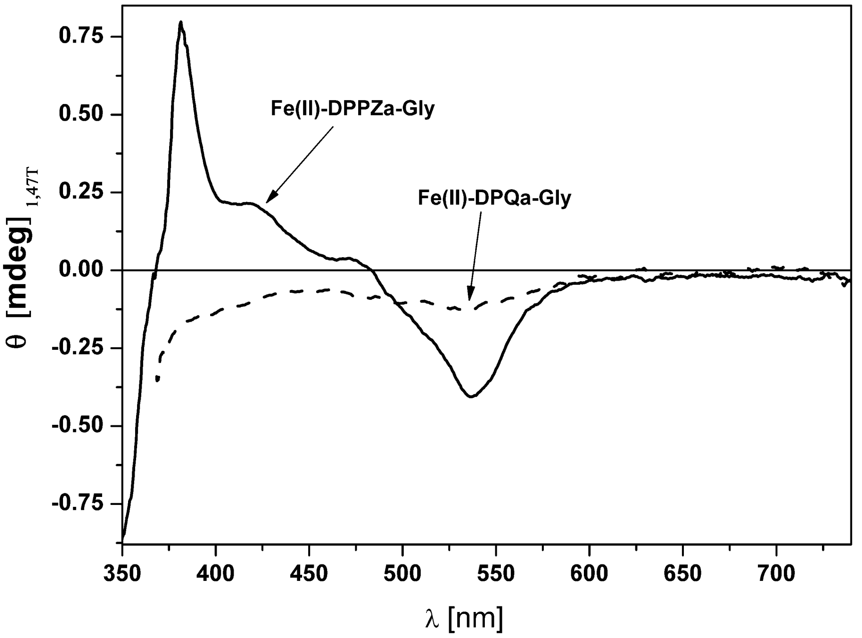

Supplementary Figure 2. Magnetic circular dichroism spectra of the Fe(II)-DPPZa-Gly and Fe(II)-DPQa-Gly complexes; concentration of metal 1 mM, metal-to-ligand molar ratio 1:3.

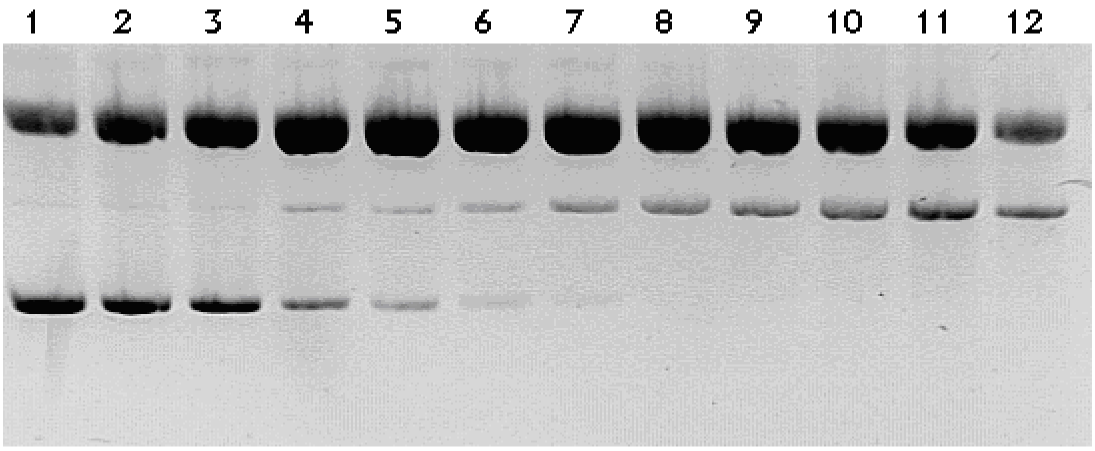

Supplementary Figure 3. Kinetics of the DNA cleavage in the presence of the Cu(II)-DFQa-Gly complex, accompanied by H_2O_2. Incubation periods: 0, 3, 5, 10, 20, 30, 45, 60, 75, 105, 135, and 180 min (lanes 1-12, respectively).

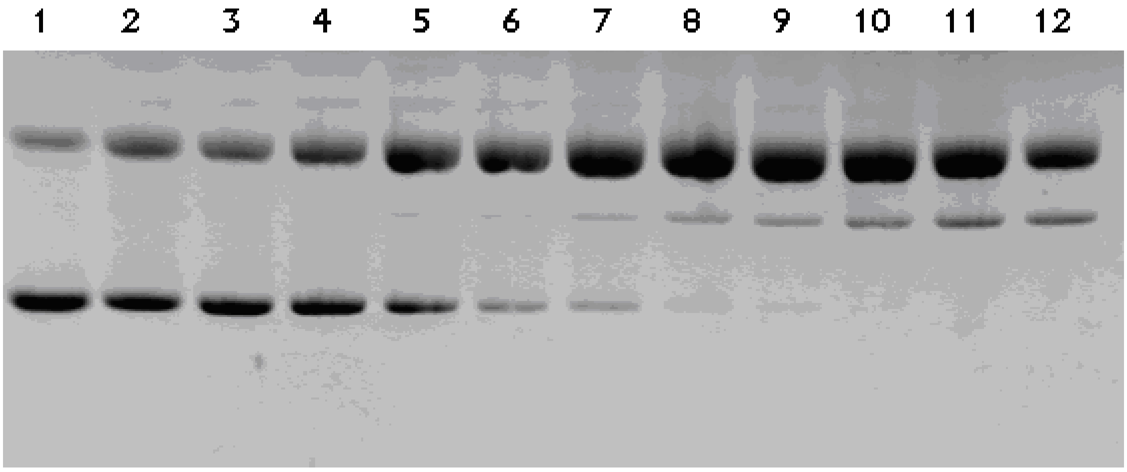

Supplementary Figure 4. Kinetics of the DNA cleavage in the presence of the Cu(II)-DPQa-Gly complex, accompanied by H_2O_2. Incubation periods: 0, 3, 5, 10, 20, 30, 45, 60, 75, 105, 135, and 180 min (lanes 1-12, respectively).

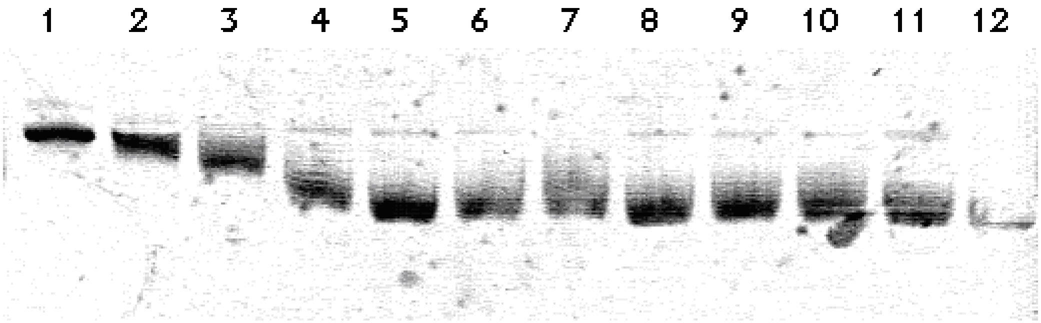

Supplementary Figure 5. Relative mobility of the plasmid DNA in the presence of DPPZa-Gly (lanes 1-6) and DPQa-Gly (lanes 7-12) at concentrations 5 mM, 1 mM, 500 μM, 100 μM, 50 μM, and 5 μM, respectively, for each compound.
